# Necrotizing myositis causes restrictive hypoventilation in a mouse model for human enterovirus 71 infection

**DOI:** 10.1186/1743-422X-10-215

**Published:** 2013-06-28

**Authors:** Jing-hui Xiu, Hao Zhu, Yan-feng Xu, Jiang-ning Liu, Xian-zhu Xia, Lian-feng Zhang

**Affiliations:** 1 Key Laboratory of Human Diseases Comparative Medicine, Ministry of Health, Beijing, China; 2 Key Laboratory of Human Diseases Animal Models, State Administration of Traditional Chinese Medicine, Institute of Laboratory Animal Science, CAMS & Comparative Medicine Centre, PUMC, Chao Yang Strict, Pan Jia Yuan Nan Li No.5, Beijing 100021, China; 3 Key Laboratory of Jilin Province for Zoonosis Prevention and Control, Institute of Military Veterinary, Academy of Military Medical Sciences, Liuyingxi Road, Building 666, Changchun City, Jilin Province 130122, China

**Keywords:** Enterovirus 71, Skeletal muscle, Necrotizing myositis, Restrictive hypoventilation

## Abstract

**Background:**

Enterovirus 71 (EV71) infections are associated with a high prevalence of hand, foot and mouth disease (HFMD) in children and occasionally cause lethal complications. Most infections are self-limiting. However, resulting complications, including aseptic meningitis, encephalitis, poliomyelitis-like acute flaccid paralysis, and neurological pulmonary edema or hemorrhage, are responsible for the lethal symptoms of EV71 infection, the pathogenesis of which remain to be clarified.

**Results:**

In the present study, 2-week-old Institute of Cancer Research (ICR) mice were infected with a mouse-adapted EV71 strain. These infected mice demonstrated progressive paralysis and died within 12 days post infection (d.p.i.). EV71, which mainly replicates in skeletal muscle tissues, caused severe necrotizing myositis. Lesions in the central nervous system (CNS) and other tissues were not observed.

**Conclusions:**

Necrotizing myositis of respiratory-related muscles caused severe restrictive hypoventilation and subsequent hypoxia, which could explain the fatality of EV71-infected mice. This finding suggests that, in addition to CNS injury, necrotic myositis may also be responsible for the paralysis and death observed in EV71-infected mice.

## Background

The human enterovirus 71 was first described by Schmidt et al. (1974). It belongs to the Picornaviridae family and has a single, positive-stranded ribonucleic acid (RNA) of approximately 7,500 nucleotides [1]. Since its discovery, there have been 13 reported outbreaks of EV71 worldwide that have resulted in a high prevalence of hand, foot, and mouth disease (HFMD) in infants and children under 6 years old [2-6]. Most infections resolve spontaneously. However, EV71 infections have occasionally caused serious, sometimes lethal, neurological complications, manifesting as aseptic meningitis, encephalitis, poliomyelitis-like acute flaccid paralysis, and neurological pulmonary edema or hemorrhage [7-10]. In recent years, numerous large outbreaks of EV71 causing HMFD have occurred in eastern and southeastern Asia [2,11-14]. No effective antiviral drugs or vaccines are available yet, and the prevention of EV71 epidemics relies solely on public surveillance.

EV71 infection-related neurological diseases and complications include encephalitis, aseptic meningitis, and brainstem encephalitis. Although many infected patients have died from a pulmonary edema or hemorrhage, results from autopsy studies suggest that the cardiopulmonary disease is neurogenic [15,16]. Pathologic analysis of several EV71-infected patients has shown that inflammation occurs in different regions of the central nervous system (CNS), including the cerebral cortex, brain stem, and all levels of the spinal cord [17]. Acute flaccid paralysis is also a notable complication associated with EV71-induced encephalomyelitis [6]. Magnetic resonance imaging (MRI) studies of infected patients have shown that EV71-induced paralysis syndromes and paralysis sites are determined by hyper intense lesions in specific sites of the anterior horns and ventral roots of the spinal cord [18]. However, the mechanism of pathogenesis during EV71 infection remains to be clarified.

Many animal models of EV71 have been used to study the virus and to develop new vaccines or other strategies to control epidemics [19-23]. After exposure to EV71, the virus establishes an infection in multiple organs and tissues in rodents, including the brain, muscles, intestines, and lungs [19-23]. Infections manifest as neutrophil vacuolation, degeneration of the anterior horn cells in the cervical cord, and extensive necrotic myositis in skeletal muscles [20,24]. However, the fatal pulmonary edema or hemorrhage seen in EV71-infected patients has not been reported in animal models.

Using a mouse model of EV71, we studied the pathogenesis of viral infection, which showed that besides brain lesions, myositis can also lead to fatality in mice.

## Results

### Muscle tropism of EV71 strains

The tropism of four clinically isolated C4 EV71 strains was analyzed in cell lines and in mice. As shown in Table 1, all four clinical strains could replicate rigorously in RD cells, but not in SHSY5Y cells. Within 48 h, the infection caused CPE in RD cells, whereas no CPE was observed in SHSY5Y cells until 7 days post infection. Similarly, when compared to brain tissue, the viral burden in muscle tissue was nearly 1000-fold higher after infection with the four clinical strains. These results suggested that muscle tropism was one characteristic of EV71 infection.

**Table 1:** The muscle tropism of four clinical isolated EV71 strains

**Strain**		**FY0805**	**JK2009**	**BZ200805**	**MZ2008**
Accession number		HQ882182	HQ694982	HQ694983	HQ694985
Log^TCID50^/ml supernatant					
RD cell	0 h	0	0	0	0
	36 h	6.63 ± 0.35^a^	6.27 ± 0.24	6.04 ± 0.42	6.88 ± 0.27
	72 h	7.97 ± 0.46	7.83 ± 0.34	7.87 ± 0.25	8.38 ± 0.36
SHSY5Y cell	0 h	0	0	0	0
	36 h	0	0	0	0
	72 h	0	0	0	0
Log^Viral RNA copies^/mg tissue					
Skeletal muscle of ICR mice	3 d.p.i	4.02 ± 0.18	4.32 ± 0.27	4.75 ± 0.33	4.32 ± 0.27
	5 d.p.i	3.87 ± 0.22	4.06 ± 0.19	4.18 ± 0.20	4.06 ± 0.19
Brain of ICR mice	3 d.p.i	1.02 ± 0.12	0.37 ± 0.12	1.19 ± 0.17	1.16 ± 0.11
	5 d.p.i	0.87 ± 0.04	0.24 ± 0.09	1.05 ± 0.23	0.98 ± 0.26

Note: a, the results were mean values of three independent experiments ± SD.

### MP10 infection caused death in 2-week-old ICR mice

**Figure 1:**
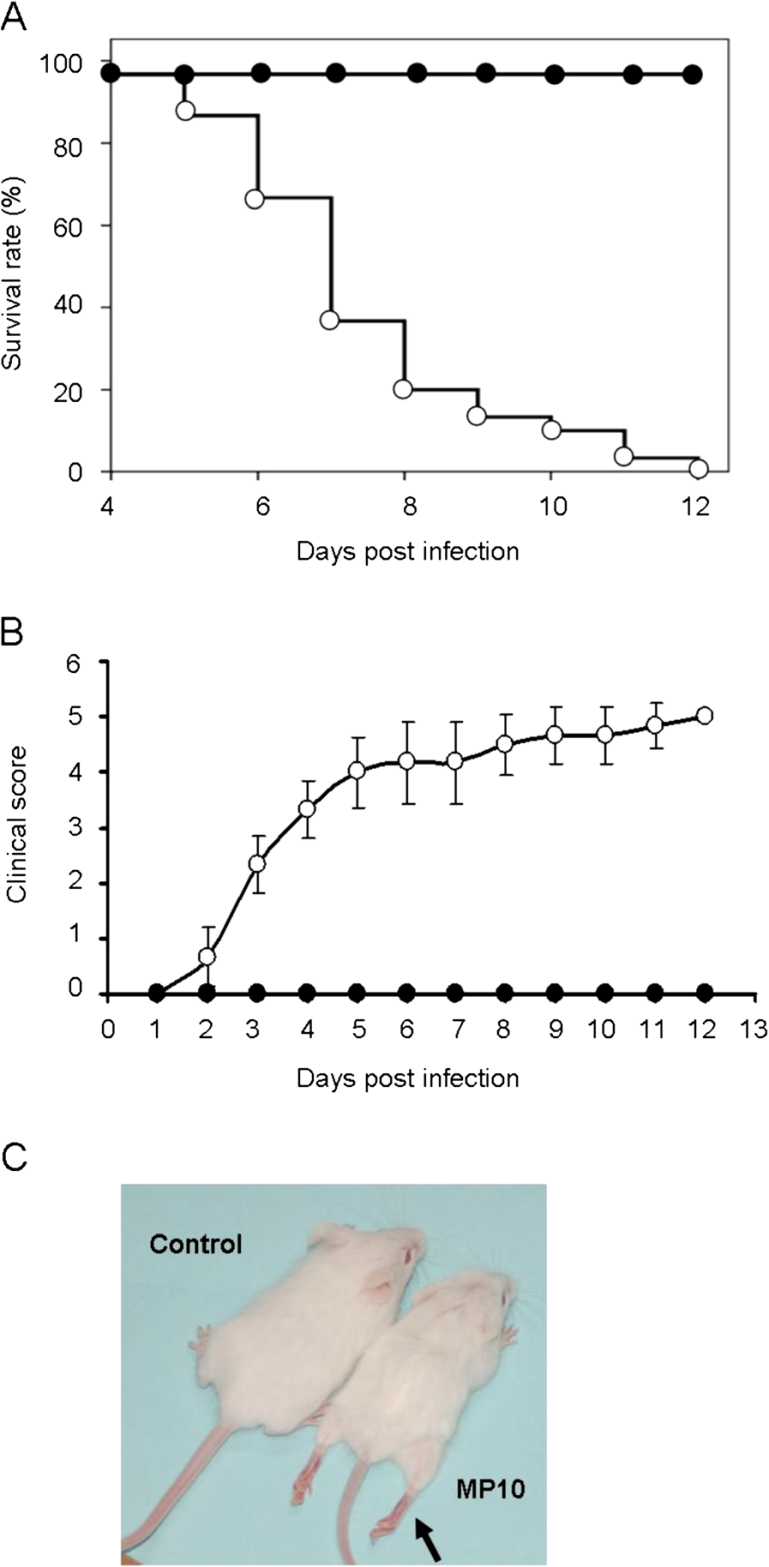
**MP10 infection causes the death of 2-week-old ICR mice within 12 days.** (**A**) Survival rates, (**B**) clinical scores, and (**C**) a photo of a typical phenotype of hind limb paralysis at 5 d.p.i. are shown (*n* = 20). (●) Mock-infected group, (○) MP10-infected group (*n* = 12). Mice in the control group were mock-infected with PBS. Paralysis of the hind limb is indicated by an arrow.

Infection by the parental strain FY0805 resulted in no symptoms in mice, whereas MP10 inoculation via intraperitoneal (i.p.) or intramuscular (i.m.) route caused typical symptoms in ICR mice under the age of 2 weeks, as reported previously [19,22]. 2-week-old ICR mice infected via i.p. or i.m. began to die at 4 d.p.i., and all of the infected mice died within 12 days (Figure 1A). Mice infected via intracranial (i.c.) showed no typical symptoms during the observation period. Mice infected via i.p. were used in subsequent studies. No skin lesions were observed in the infected mice during the 14-day observation period, although other signs of infection included weight loss, a humped posture, ruffled fur, and progressive paralysis (Figure 1B and C). Furthermore, the mice failed to open their eyes for 4 days after MP10 infection, and they showed tachypnea and heavy breathing in the moribund state.

### Virus replicated in skeletal muscle and caused necrotizing myositis

**Figure 2:**
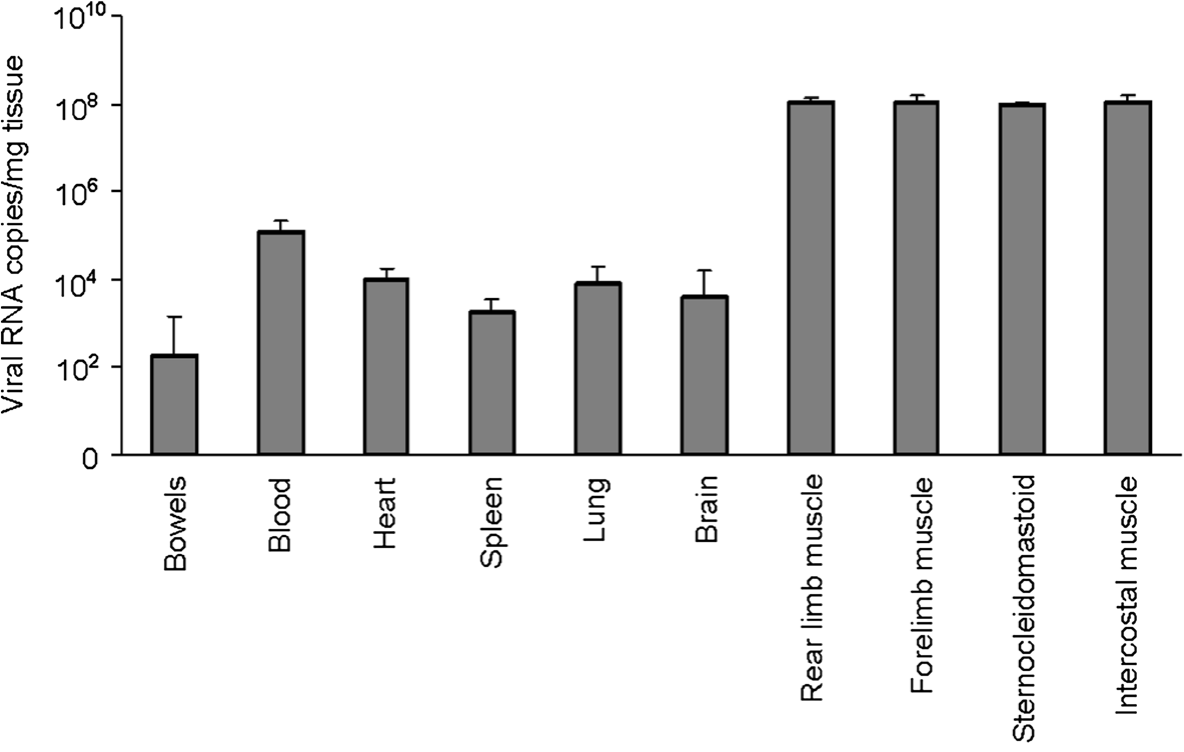
**MP10 mainly replicates in the skeletal muscle tissues of infected mice.** Viral RNA copies in tissues of EV71-infected mice were determined by qRT-PCR, at 3 d.p.i. (*n* = 20).

Tissue samples from infected mice were subjected to virus burden determination by quantitative real-time polymerase chain reaction (qRT-PCR) during the observation period. The viral RNA replication reached a peak at 3 d.p.i. (Additional file 1: Figure S1). At this time point, abundant viral RNA was detected in skeletal muscle tissues, which included both the rear limb and fore limb muscles, the sternocleidomastoid muscle, and the intercostal muscles. Because viral RNA copy numbers in the skeletal muscle tissues were observed to be at least 1000-fold higher compared to other tissues (Figure 2), we concluded that the MP10 virus exhibits muscle tropism by preferentially replicating in skeletal muscle tissues. Consequently, EV71 infection resulted in severe necrotizing myositis in infected skeletal muscle tissues.

**Figure 3:**
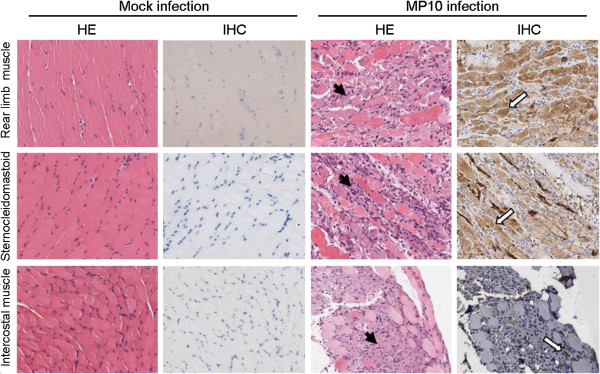
**MP10 replication causes severe necrotizing myositis in the skeletal muscle tissues of mice.** The pathology and viral distribution in tissues of MP10-infected mice (from 3 d.p.i. to 9 d.p.i., *n* = 6) were observed by HE stain (left panel) and IHC (right panel). Representative images from the rear limb muscle, sternocleidomastoid muscle, and intercostal muscle at 5 d.p.i. are shown, and the positions of lesions and viral antigens are denoted with solid and open arrows, respectively. The results of mock-infection were used as control. Magnification: 100 ×.

We examined the rear limb muscles, the sternocleidomastoid muscles, and the intercostal muscles by hematoxylin and eosin staining (HE) staining, during the observation period. All three tissues presented necrotizing myositis from 3 d.p.i. (Figure 3A, C and E), accompanied by abundant viral antigen distribution, as detected by immunohistochemical staining (IHC) (Figure 3B, D and F). Although severe pathological changes occurred in the skeletal muscle, no typical lesions associated with EV71 infection were observed in other tissues, such as the brain or spinal cord (data not shown). Furthermore, viral antigens were not detected by IHC in tissues other than the skeletal muscle, which indicated that the concentration of the virus was lower than the detection limit (data not shown).

### MP10-infected mice exhibited severe restrictive hypoventilation and hypoxia

**Figure 4:**
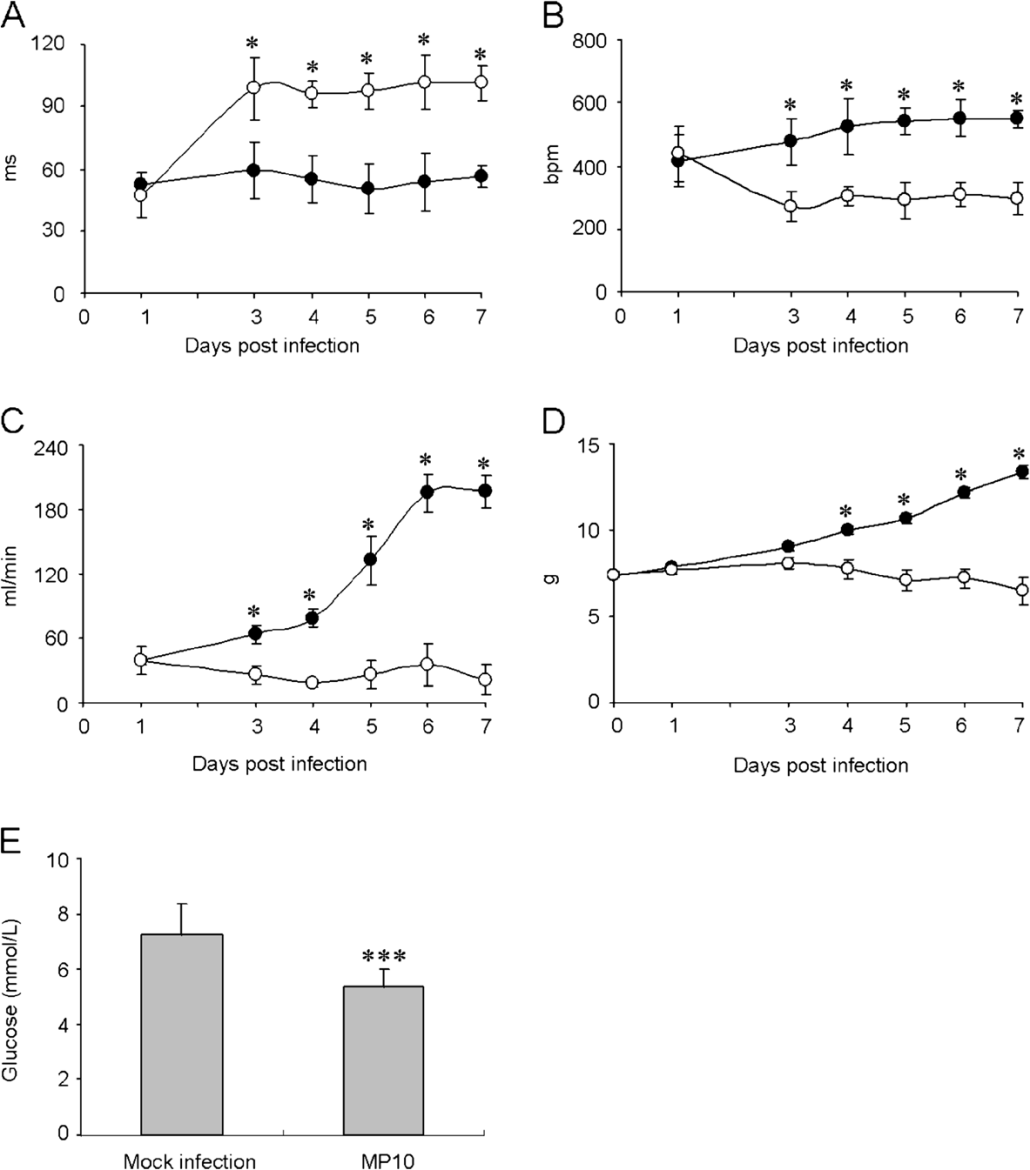
**MP10 infection causes severe restrictive hypoventilation.** Parameters reflecting respiratory functions, including (**A**) inspiratory time (TI), (**B**) respiratory frequency (F), and (**C**) minute volume (MV) of mock-infected and MP10-infected mice were recorded for 7 days (*n* = 20). Variations in body weight (**D**) were recorded for normalization. (●) Mock-infected group, (○) MP10-infected group (*n* = 12). **E**, the glucose levels in the blood of mock-infected and MP10-infected mice were detected at 6 d.p.i. (*n* = 24). * *P* < 0.05 and *** *P* < 0.001.

Because the muscle tissues related to respiratory function were necrotic, and the infected mice showed tachypnea and heavy breathing in the moribund state, we tested whether the respiratory function of the infected mice was affected due to the dysfunction of their respiratory-related muscles. Compared to the mock-infected mice, the parameters of respiratory function in the infected mice were significantly changed at 3 d.p.i. For example, the inspiratory time was significantly extended, which caused a drop in respiratory frequency (Figure 4A and B). Furthermore, the tidal volume and expiratory volume of respiration in infected mice were significantly reduced compared to those in mock-infected mice (Additional file 1: Figure S2). The obvious reduction in the respiratory minute volume indicated that severe restrictive hypoventilation and respiratory depression had occurred, accompanying the weight loss observed in MP10-infected mice from 3 d.p.i. (Figure 4C and D).

High glucose levels have previously been shown to be related to pulmonary edema in a clinical setting [25]. Therefore, the blood glucose levels of the infected mice were determined. The results showed that, in contrast to the high glucose levels in blood from patients who developed pulmonary edema, the glucose concentration in the blood from infected mice was significantly reduced by 26% of that of the blood glucose concentration in control mice at 6 d.p.i. (Figure 4E), This result indicated that the severe restrictive hypoventilation observed in the infected mice was not caused by pulmonary edema.

**Figure 5:**
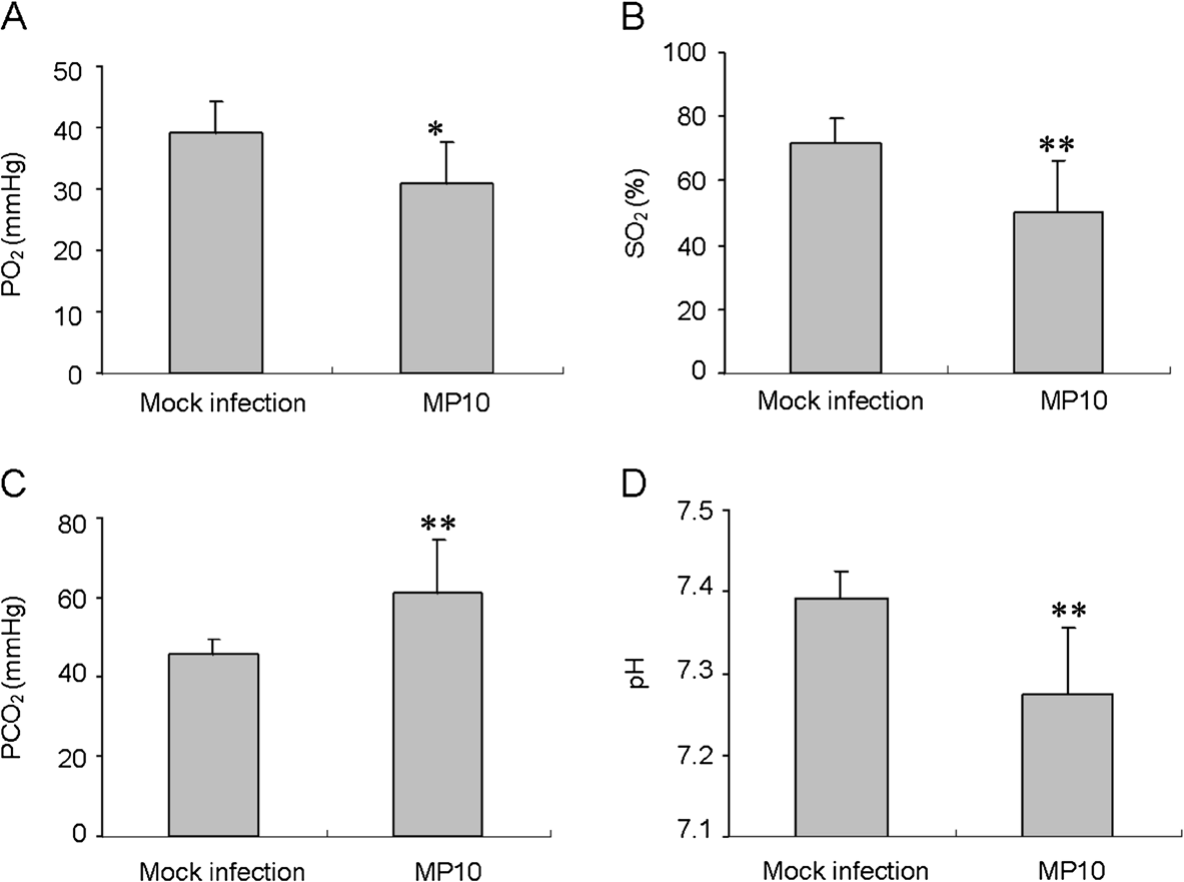
**MP10 infection causes a change of parameters in the blood of infected mice.** Levels of (**A**) PO_2_, (**B**) SO_2_, and (**C**) PCO_2_ in the blood, and (**D**) the pH of the blood in mock-infected and MP10-infected mice were determined at 6 d.p.i. (*n* = 24). * *P* < *0.05* and ** *P < 0.01*.

The effect of respiratory depression on the infected mice was also determined. In the infected mice, PO_2_ and SO_2_ were reduced by 20% and 30% respectively, at 6 d.p.i. This result indicated a hypoxic state at later stages of infection (Figure 5A and B). The restriction of oxygen caused a significant enhancement of PCO_2_ (33%) and reduced the pH of the blood of infected mice (Figure 5C and D). These results demonstrated that severe respiratory depression resulted in hypoxia in the infected mice.

## Discussion

In this study, we have demonstrated that skeletal muscle (rear limb muscle, fore limb muscle, sternocleidomastoid muscle, and intercostal muscle) is the main tissue supporting EV71 replication in mice. We showed that extensive necrotic myositis induced severe restrictive hypoventilation and significantly lower oxygen concentrations in the blood, which may explain the fatalities observed in EV71-infected mice.

Most autopsy reports from EV71-infected patients with fatal complications have shown that EV71 was mostly found in brain tissue, although viral antigens were also detected in the lungs and intestines [16,26-28]. Therefore, EV71 is recognized as a medically important neurotropic enterovirus, as the poliovirus has been nearly eradicated in most countries around the world [29]. EV71 infections can cause inflammation in the CNS, which can lead to severe neurological diseases, including aseptic meningitis, brain stem encephalitis, and pulmonary edema [16]. These serious neurological complications are thought to be the cause of death for those affected by EV71 infections [30].

Similar to infected patients, EV71 replication in the brain tissues of animals leads to neuron loss, neuronal degeneration, and inflammation in the CNS [19,31]. In EV71-infected monkeys, the neurological manifestations include flaccid paralysis, tremors, and ataxia, whereas the more severe symptoms in infected mice are paralysis of the hind limb and death [19,31]. However, in spite of the neurotropism of EV71 observed in patients, the virus has frequently been found in the organs of many non-neural systems, including the blood, heart, lungs, spleen, and muscles. In addition, in murine and cynomolgus monkey animal models, the viral load in muscle tissues was significantly higher than that in brain tissues, a finding that has not been reported in humans [20,32,33].

In the present study, we observed a muscle tropism of EV71, which appears to be a characteristic of clinical strains of the virus (Table 1). After inoculating the virus in ICR mice, viral copies in muscle tissue were at least 10,000-fold higher than in other tissues, such that the infection caused severe necrotic myositis in skeletal muscle tissues (Figures 2 and 3). The necrotic myositis caused by EV71 infection has been documented in previous studies and has been proposed to be one of the reasons for the flaccid paralysis of limbs in infected mice [20]. Furthermore, MP10 showed lower tropism in neurological tissues compared to previous studies, and no typical lesions in brain tissues were observed postinfection. These results, together with the observation that infection with MP10 via i.c. caused no typical symptoms in mice, strengthen the argument that the paralysis in MP10-infected mice was caused by necrotic myositis in the muscle, rather than lesions in the CNS.

Neurological cardiopulmonary dysfunction is believed to be a serious complication in clinical patients that can sometimes lead to death [10,34]. Although most animal models of EV71 infection have demonstrated viral infection and pathology in the CNS, only a minority developed pulmonary edema. Liu et al. described a neonatal monkey model of EV71 infection via i.c., in which injury to the CNS induced pulmonary edema [35]. Huang et al. reported that EV71-infected mice exhibited pulmonary edema and hemorrhage. However, the pulmonary edema was induced by systematic symptoms, such as cytokine infiltration or inflammation, rather than lesions in the CNS [36]. We demonstrated that the ventilation function of infected mice was significantly impaired compared to the control (Figure 4), which caused lower oxygen concentrations in the peripheral blood (Figure 5). Because no typical lesions in the brain tissue or lungs were observed in infected mice, we hypothesized that the severe restrictive hypoventilation was caused by myositis in the respiratory-related skeletal muscles, leading to fatal hypoxia. Clinical studies on the pathology of EV71-infected patients have focused on neurological tissues and on organs other than muscle tissues [16,17,26,28]. Our study suggests that the infection and pathology of the virus in skeletal muscles should be monitored more closely in the clinic.

## Materials and methods

### Cells and viruses

Human rhabdomyosarcoma cells (RD) for viral propagation were maintained in Dulbecco’s Modified Eagle Medium (DMEM) containing 10% fetal bovine serum (FBS), as previously described [37]. Four C4 EV71 strains were isolated from patients in China in 2008 by two passages, with the clinical specimens purified in RD cells (Table 1). To prepare the mouse-adapted virus, serial passages were initiated by an intraperitoneal (i.p.) inoculation of 5 × 10^7^ TCID_50_ of the FY0805 strain into 10-day-old imprinting control region (ICR) mice (*n* = 6). The skeletal muscle from the limbs of the infected mice was harvested and pooled 3 d.p.i. A homogenate suspension (20% wt/vol) was prepared with DMEM, and approximately 200 μl of the supernatant was i.p.-inoculated directly (the viral stock was not amplified in cell culture between the mouse passages) into each mouse of a new mouse litter for the second and subsequent passages. A mouse-adapted viral strain derived from the 10th passage was plaque-purified twice in RD cells and designated as MP10 (Accession number: HQ712020). Working stocks containing 10^9^ TCID_50_ per ml were prepared for infection experiments.

### Virus inoculation

The mice used in the present study were provided by the Institute of Laboratory Animal Science, Peking Union Medical College, and the animal protocols were approved by the institutional animal care and use committee (GC-09-2077). Unless otherwise noted, groups of ten 2-week-old mice were infected with virus via i.p., i.m. or i.c. injection. The viral dose for mouse challenge was 2 × 10^6^ TCID_50_ per gram of weight, without specification. Mock-infected mice were given phosphate-buffered saline (PBS) (pH = 7.4) and kept in isolated cages.

### Viral tropism test

The tropism of EV71 strains was tested on cell lines and in ICR mice. To analyze the tropism of the virus on cells, RD cells or human neuroblastoma cells, SHSY5Y, (2 × 10^4^ cells/well) were plated in 96-well plates with DMEM media without antibiotics and grown overnight at 37°C. Cells at > 90% confluency were infected with 100 TCID_50_ of virus in DMEM media (containing 2% FBS). The medium was removed at 2 h postinfection, the cells were washed three times with PBS, and fresh DMEM media containing 2% FBS was added to the cells. The supernatant was sampled at different time points for titer determination of the RD cells. To analyze the tropism of the virus on the mouse tissues, groups of six 10-day-old ICR mice were inoculated with 1 × 10^7^ TCID_50_ of virus. The tissues were sampled at different time points and sent for viral RNA analysis by qRT-PCR.

### Clinical scores

The MP10-infected mice were observed twice daily for up to 14 days for the clinical symptoms of weight gain or loss, and mortality. A clinical score was assigned, as previously described [38], as follows: 0, healthy; 1, ruffled hair; 2, weakness in the hind limbs; 3, paralysis in a single hind limb; 4, paralysis in both hind limbs; and 5, death.

### Determination of the virus burden

qRT-PCR was used to detect viral RNA copies in the mouse tissues postinfection, as described previously, with minor modifications [39]. Briefly, total RNA was isolated from 30 mg of tissue using TRIzol reagent. The isolated RNA was reverse transcribed with random hexamers using a reverse-transcription kit (Promega, China), and the viral cDNA was assayed by PCR amplification for FY0805 (nucleotides 2462–2635) with the following primers: EV71-S1 (5′- AGATAGGGTG GCAGATGTAA TTGAAAG-3′) and EV71-A1 (5′- TAGCATTTGA TGATGCTCCA ATTTCAG -3′). The incorporation of the SYBR fluorescent dye into the DNA double-strand was monitored and analyzed using a Roche Light Cycler 3.5 system. A DNA fragment with known copies was used as the standard to calculate the copy number of viral RNA in the infected tissues. The results were normalized to GAPDH.

### Pathology

For each experimental group, six mice were subjected to pathologic examination from 3 d.p.i. After euthanasia, brain and muscle of mouse tissues were immediately immersion-fixed in 10% buffered formalin for 48 h. Different parts of the tissues were bisected, embedded in paraffin, and stained with the HE stain. Ten sections of each tissue were observed per animal in a blinded manner. The histopathology of skeletal muscle was evaluated based on the following three parameters: inflammation, muscle fiber degeneration, and necrosis.

### Immunohistochemical staining

Immunohistochemical (IHC) staining was used to detect viral antigens in the tissues of infected mice. IHC was performed as previously described [37] on formalin-fixed tissue sections. An EV71 monoclonal antibody against VP1 (1:200 dilution, Millipore, USA) was used as a primary antibody. The sections were washed three times with PBS and incubated with horse radish peroxidase (HRP)-conjugated goat anti-mouse IgG (1:5000 dilution, Sigma, Germany) for 1 h at 37°C. The sections were developed with 3-3′ diaminobenzidine (DAB) and viewed with a light microscope.

### Respiratory function assessment

Ventilatory function was assayed using a barometric whole-body plethysmograph. Unanesthetized, unrestrained mock-infected and MP10-infected mice were placed in a clear plexiglass chamber (Buxco, Wilmington, NC) [40]. After calibration of the each chamber, one mouse was placed in a small plexiglass container within each plethysmograph chamber, and measurements were made over a period of 5 min. Prior experiments have established that reproducible results are obtainable within this time period. Because recordings were made over short time periods, box humidity and temperature levels were set at the humidity and temperature levels recorded within the same room. The instrument settings for animal body temperature were set at 37.5°C, and bias flow was set at 0.5 L/min. Parameters such as frequency, inspiratory time (TI), respiratory frequency (F), minute volume (MV), tidal volume (TV), and expiratory volume (EV) were measured and analyzed using the method of Drorbaugh and Fenn and recorded using the BioSystem XA software (Buxco,USA).

### Blood gas analysis

Arterial blood was sampled from the neck of infected mice at 6 d.p.i. The pH, PO_2_, PCO_2_ and SO_2_ content of the blood were analyzed using a handheld i-STAT analyzer (Abbott, USA) with a G3+ cartridge, according to the manufacturer’s protocol. Blood glucose levels were measured using a glucometer (GlucoTrend®, Roche Diagnostics, Burgdorf, Switzerland).

### Statistical analysis

All data are expressed as the mean ± SD. Data obtained from all experiments were analyzed by *t*-test. A *P*-value of < 0.05 was considered to be significant.

## Consent

Written informed consent was obtained from the patient’s guardian/parent/next of kin for the publication of this report and any accompanying images.

## Abbreviations

HFMD: Hand, foot and mouth disease; CNS: Central nervous system; EV71: Enterovirus 71; MRI: Magnetic resonance imaging; RD: Human rhabdomyosarcoma cells; DMEM: Dulbecco’s Modified Eagle Medium; TI: Inspiratory time; F: Respiratory frequency; MV: Minute volume; TV: Tidal volume; EV: Expiratory volume; d.p.i.: days post infection; IHC: Immunohistochemical; HRP: Horse radish peroxidase; ICR: Institute of Cancer Research; qRT-PCR: quantitative real-time polymerase chain reaction.

## Competing interests

The authors declare that they have no competing interests.

## Authors’ contributions

JHX carried out the quantitative assays. JNL participated in cell culture and preparation. JNL expanded EV71using the viral plaque assay. JHX and YFX carried out tissue staining and imaging. JNL performed data analysis and designed the primers for RT and qPCR. XZX, LFZ and JNL conceived and designed the study and prepared the first draft of the manuscript. HZ carried out the MRI on mice. All authors read and approved the final version of this manuscript.

## Supplementary Material

Additional file 1 **Figure S1.** EV71 replication curve in skeletal muscle. **Figure S2**. MP10 infection caused severe restrictive hypoventilation. Click here for file
