# Supplementary material for: Necrotizing myositis causes restrictive hypoventilation in a mouse model for human enterovirus 71 infection
Source: Virol J. 2013 Jun 28;10:215. doi: 10.1186/1743-422X-10-215 (PMC3710232; doi:10.1186/1743-422X-10-215)
Supplement: Additional file 1 — Figure S1. EV71 replication curve in skeletal muscle. Figure S2. MP10 infection caused severe restrictive hypoventilation. [file 1743-422X-10-215-S1.doc]

**Supplemental Figure Legends.**

**Supplemental Figure 1. EV71 replication curve in skeletal muscle.** The viral RNA copies were determined by qRT-PCR in muscle tissues of MP10-infected mice (n=12 at each time point).

**Supplemental Figure 2. MP10 infection caused severe restrictive hypoventilation.** The parameters reflecting respiratory functions, including: (A), tidal volume (TV), and (B), expiratory volume (EV) of mock-infected and MP10-infected mice were recorded for 7 days period. (●) Mock-infected group, (○) MP10-infected group (n=12), * *p<0.05*.

**
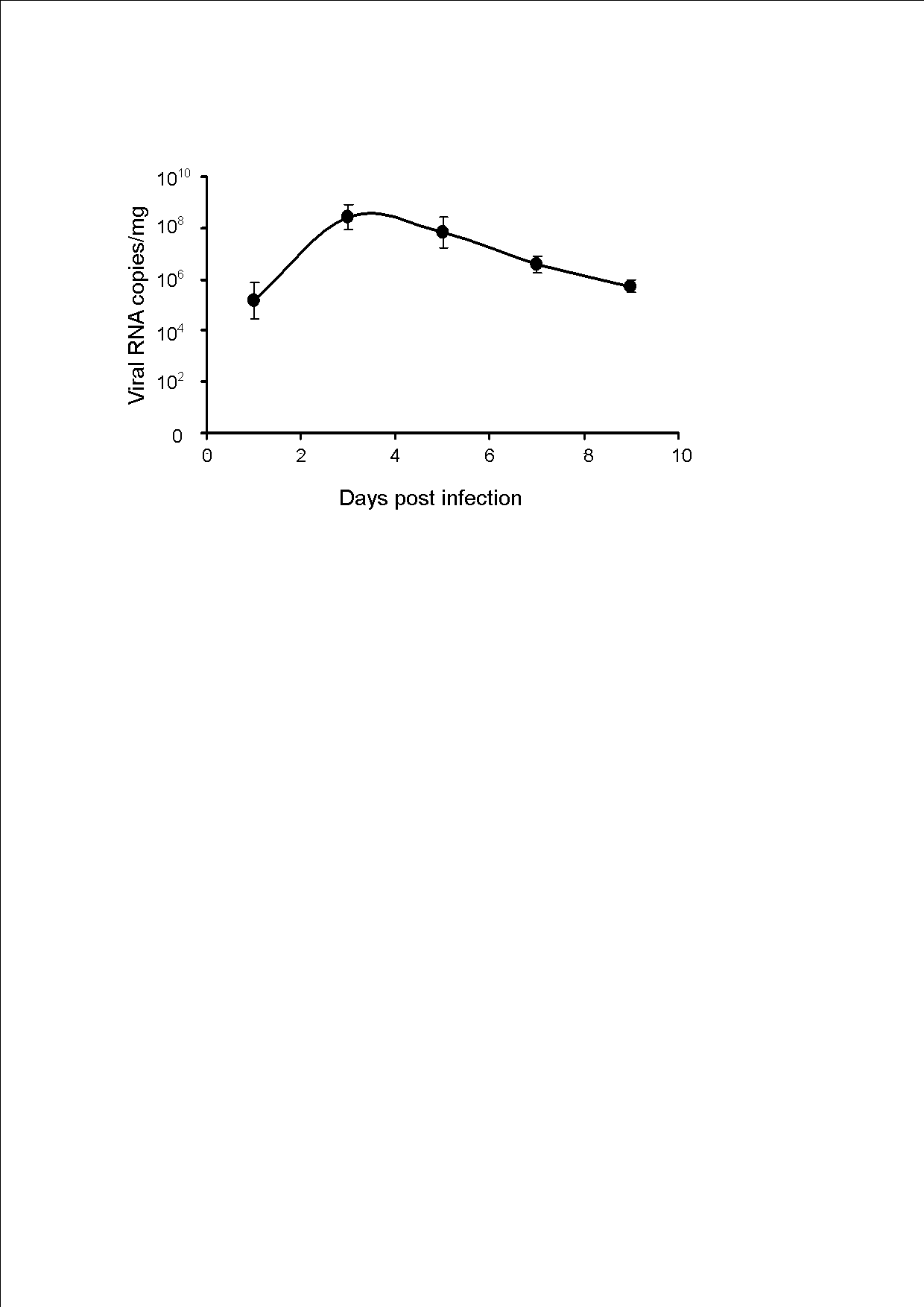
**

**Supplemental Figure 1.**

**
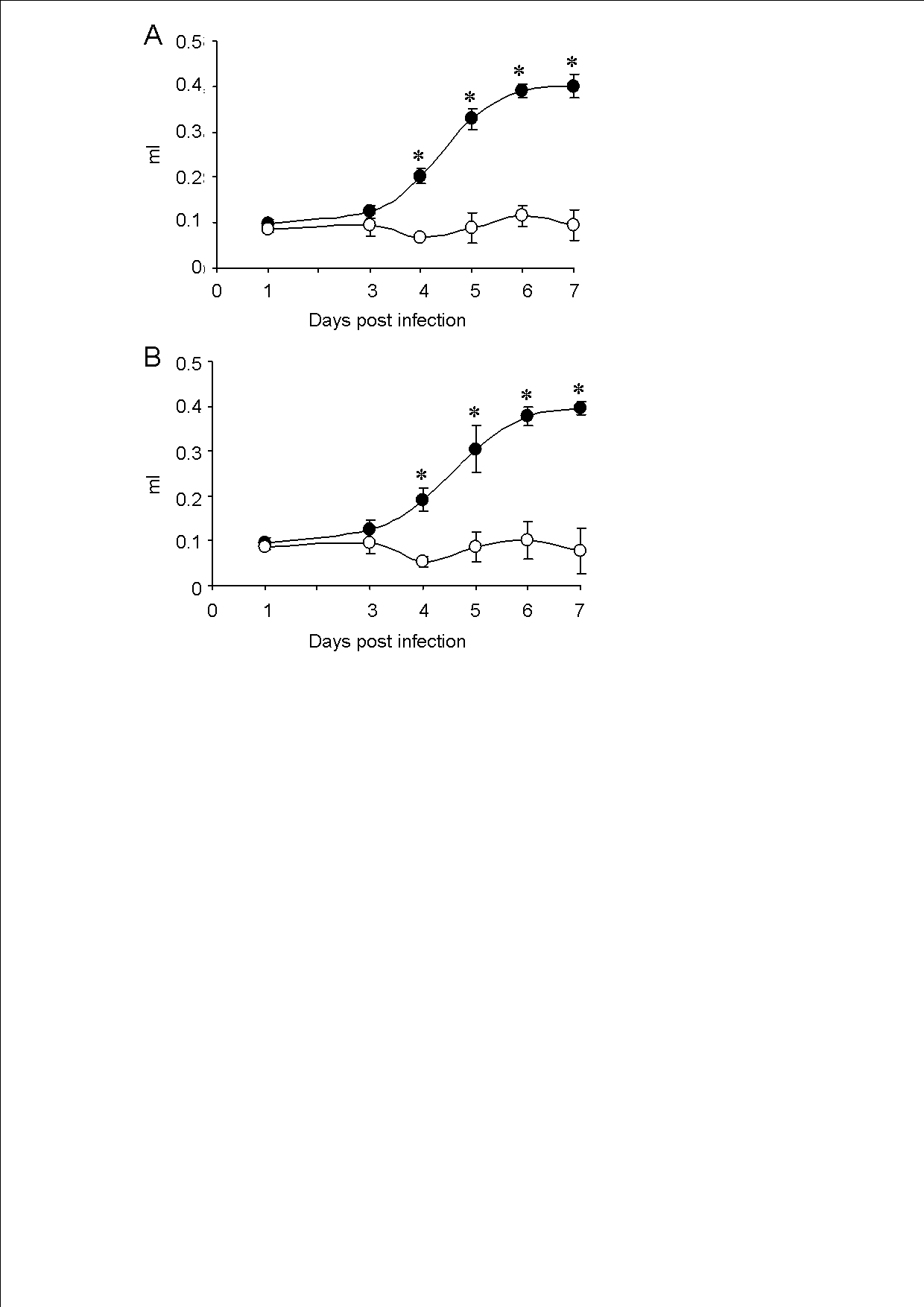
**

**Supplemental Figure 2.**
